# Sexual harassment, sexual violence and subsequent depression and anxiety symptoms among Swedish university students: a cohort study

**DOI:** 10.1007/s00127-024-02688-0

**Published:** 2024-06-26

**Authors:** Fred Johansson, Klara Edlund, Jorunn Sundgot-Borgen, Christina Björklund, Pierre Côté, Clara Onell, Tobias Sundberg, Eva Skillgate

**Affiliations:** 1 Department of Health Promotion Science, Sophiahemmet University, Box 5605, Stockholm, 114 86 Sweden; 2 Department of Clinical Neuroscience, Karolinska Institutet, Stockholm, Sweden; 3 Department of Sport Medicine, Norwegian School of Sport Sciences, Oslo, Norway; 4 Unit of Intervention and Implementation Research for Worker Health, Institute of Environmental Medicine, Karolinska Institutet, Stockholm, Sweden; 5 Institute for Disability and Rehabilitation Research, Faculty of Health Sciences, Ontario Tech University, Oshawa, ON Canada

**Keywords:** Sexual harassment, Sexual violence, Depression, Anxiety, Students, Cohort study

## Abstract

**Purpose:**

To determine the gender-specific impact of recent exposure to different forms of sexual harassment and sexual violence (SHV) on depression and anxiety symptoms three, six, and nine months later.

**Methods:**

We recruited 2229 women and 1274 men studying at Swedish universities and followed them with web-surveys every three months over one year. We estimated mean differences (MDs) of depression and anxiety symptoms between exposed and unexposed at each follow-up, adjusting for prior SHV, prior depression and anxiety symptoms and potential confounders.

**Results:**

For women, sexual harassment (wide subjective definition) was associated with higher symptom levels of depression (MD 1.0 [95% CI: 0.3; 1.7]) and anxiety (MD 0.8 [95% CI: 0.3; 1.4]) three months later. Unwanted sexual attention was associated with higher symptom levels of anxiety three (MD 0.5 [95% CI: 0.1; 0.8]) and six months later (MD 0.4 [95% CI: 0.0; 0.7]). Exposure to sex against ones will was associated with higher depression symptoms three (MD 1.7 [95% CI: 0.1;3.4]), and six months later (MD 3.1 [95% CI: 1.0; 5.2]). Trends indicated that associations with subsequent mental health differed between forms of SHV among women, and that most associations were more pronounced in temporal proximity to the exposures. For men, we refrain from interpreting the results since they showed high variability and were not robust to sensitivity analyses using multiple imputation to account for missing outcome data.

**Conclusions:**

Among women, several forms of SHV were associated with higher subsequent depression and anxiety symptoms.

**Supplementary Information:**

The online version contains supplementary material available at 10.1007/s00127-024-02688-0.

## Introduction

Sexual harassment and sexual violence are often conceptualized as a continuum of negative sexual experiences of varying severity, ranging from sexualized jargon and jokes to sexual abuse and rape [1–4]. Sexual harassment is a common experience among university students that disproportionally affect women [1, 5, 6]. There are also reports of high rates of sexual violence in universities, with about 25% of women and 6% of men undergraduate students experiencing non-consensual sexual contact while enrolled at university [7]. In this study, we refer to the continuum of negative sexual experiences as sexual harassments and sexual violence (SHV).

Cross-sectional studies suggest that experiences of sexual harassment is associated with elevated levels of psychological distress [8–10] and negative emotional states such as depression, anxiety and loneliness [11] among university students. Cohort studies report that experiencing sexual harassment is also associated with higher subsequent levels of psychological distress, and substance use problems among university students [12–14].

For many victims, exposure to SHV is no one-time event, but rather a series of recurrent negative experiences [15]. Exposure to SHV at a given time-point is associated with both previous SHV and previous mental health problems [12, 16–18]. Exposure to SHV at any single time-point may therefore serve as a proxy for prior levels of SHV which may also be affected by prior mental health. To identify the impact of *recent* exposure to SHV on mental health, one must therefore control for both prior SHV and prior mental health [14, 19]. With adjustment for prior exposure, estimates may be interpreted as the effect of recent SHV experiences on mental health, rather than the accumulated effect of repeated SHV. Estimating the effect of recent SHV is of interest because it may more closely mirror the effect of intervening to reduce SHV here and now, and is subject to fewer biases than analyses not adjusting for prior exposure [19, 20]. Several cohort studies report associations between SHV and subsequent mental health, after controlling for prior mental health symptoms [12–14, 21]. To our knowledge, only one study has shown that associations between SHV and depression and anxiety remain after controlling for both prior levels of SHV and depression and anxiety [14], but this study had no control for other potential confounders. In order to strengthen the evidence of causal effects of SHV on mental health, there is a need for more studies controlling for both prior mental health and SHV [19]. Further, although exposure to SHV may persist over time, little is known of the duration of the potential effects of SHV on mental health.

Available evidence suggests that SHV is associated with mental health problems [8–14, 21–23], but less is known about how *specific forms* of SHV are related to mental health problems. This is primarily due to the fact that most previous studies have combined different forms of SHV into broad exposure categories [8, 9, 21]. While providing information on overall associations between SHV and mental health, this approach may obscure differences in mental health impact between different forms of SHV. Since SHV is conceptualized as a continuum of sexual experience of *varying severity*, it seems plausible that the mental health impact may differ between different forms of SHV. There are some studies pointing in this direction. For instance, sexual coercion show stronger association to mental health problems, than does sexual harassment in general, in both cross-sectional [24, 25] and cohort studies [12]. Because the SHV continuum includes negative sexual experiences of varying severity, we believe there is a need to differentiate between different forms of SHV and their potential impact on mental health problems among university students.

In this cohort study, we aimed to investigate the impact of recent exposure to different forms of SHV; (1) unwanted sexual attention, (2) offensive sexual remarks, (3) presentation or distribution of sexist material, (4) uncomfortable touching, (5) being offered benefits for sex and (6) sex against ones will, along with a wide definition of sexual harassment: sexual harassment (wide subjective definition) on levels of depression and anxiety symptoms three, six and nine months later, for women and men, respectively.

## Methods and material

### Source population

We used data from the Sustainable UNiversity Life (SUN) cohort study, described in detail in the study protocol [26]. Eligible participants were undergraduate or graduate (including up to masters’ level) full-time students at eight universities in and around Stockholm and Örebro with at least one academic year left until graduation. The targeted universities constitute a convenience sample, aiming to represent a variety of different university programmes.

### Study sample, recruitment, and data collection

Eligible students were informed about the study during in-class presentations by study staff and/or by an email with a link to the first web-survey. Students who agreed to participate were followed every three months for one year with web-surveys, giving a total of five time-points of data collection. Data collection was ongoing between August 19, 2019, and December 15, 2021. For each survey that the participants filled out, they received a one-month free pass at a health club.

**Fig. 1:**
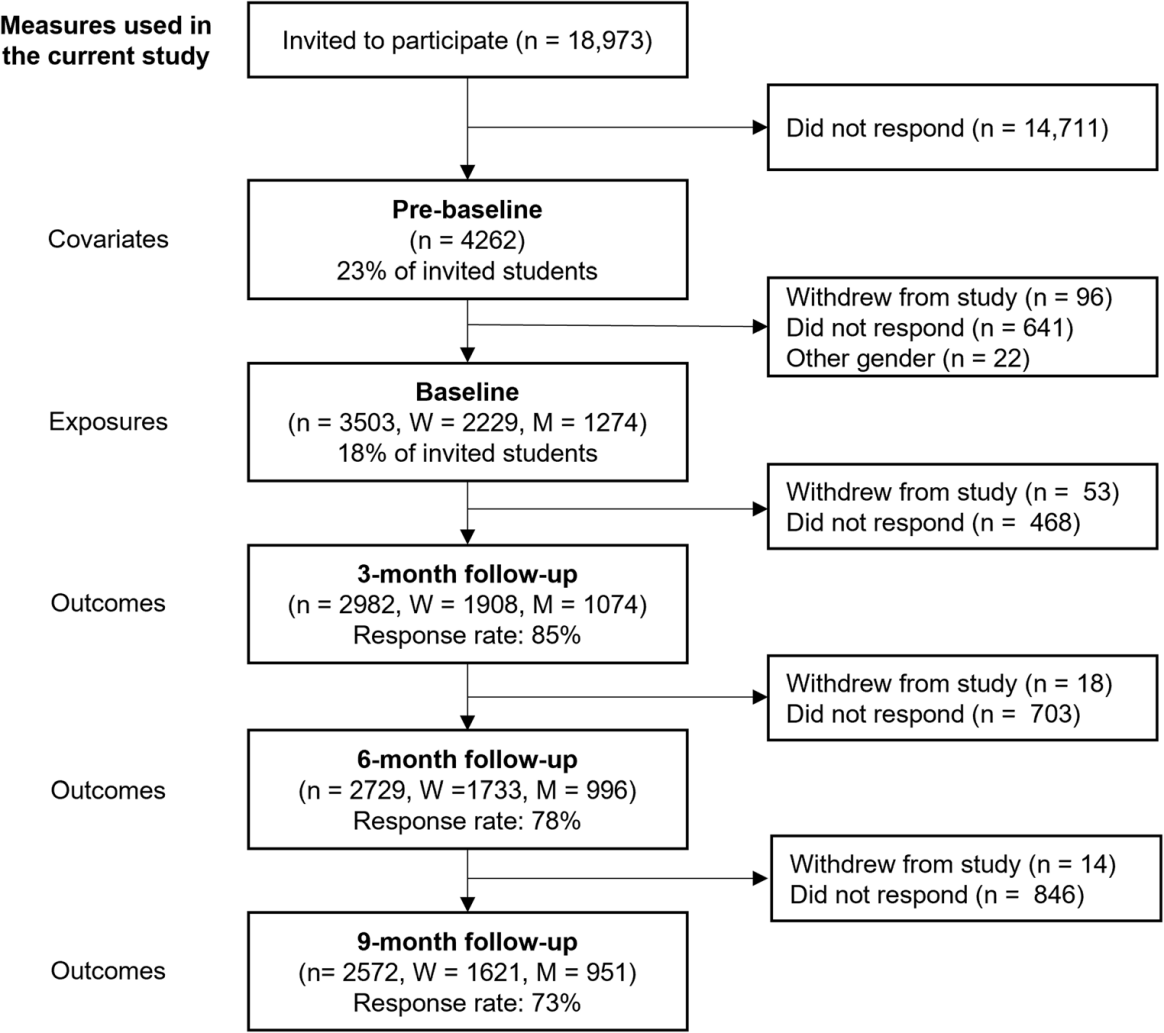
Flowchart of the inclusion of participants. Response rates are presented in relation to the baseline time-point. Participants were allowed to respond to later surveys even if they had missed earlier surveys and non-responders at each follow-ups are presented as number of persons not responding in relation to the baseline time-point. M = Men, W = Women

In the current analysis, we refer to the first time-point of data collection as pre-baseline, which was used for the measurements of potential confounders. The second time-point is referred to as baseline and used for exposure measurements. The third, fourth and fifth time-point, referred to as the 3-month, 6-month, and 9-month follow-up (FU3, FU6, FU9) with the figures representing months since baseline, were used for outcome measurements (Fig. 1). The study includes all women and men participating in the SUN cohort study and responding at baseline (*n* = 3503). Of those, 85% (*n* = 2982) responded at FU3, 78% (*n* = 2729) at FU6 and 73% (*n* = 2572) at FU9. Participants stating a gender identity other than woman or men were excluded from the study (*n* = 22) (Fig. 1).

### Measures

#### Exposures

**Fig. 2:**
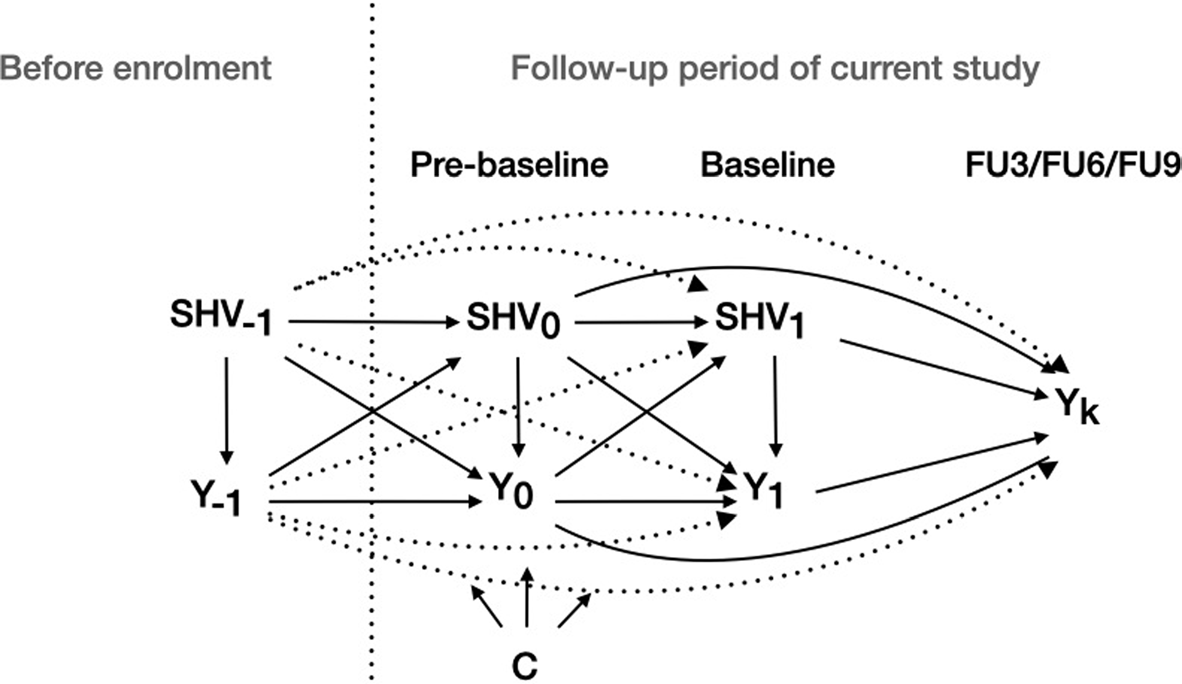
Directed acyclic graph showing the assumed causal structure between sexual harassment and violence (SHV) and the outcomes (depression and anxiety symptoms) at different timepoints before and during the follow-up period. Y_k_ denotes the outcome at follow-up *k* (FU3, FU6, FU9). SHV_t_ denotes value of the exposure to SHV at time *t* (before enrolment, pre-baseline, baseline). C is a set of pre-baseline covariates including age, place of birth, parental education, type of education, study year, education type and civil status. The dashed arrows represent potentially biasing paths of direct long-term effects (not mediated through intermediate variables) that were not adjusted for in current analysis but assumed to be of little or no influence

SHV was measured at both pre-baseline (SHV_0_ in Fig. 2) and baseline. (SHV_1_ in Fig. 2). The recall period was one year at pre-baseline and three months at baseline (indicating if they had experienced sexual harassment since pre-baseline). Baseline measures were used as the exposures and pre-baseline measures as covariates.

##### Sexual harassment (wide subjective definition)

was measured by letting participants read the statement “*According to the Discrimination Act, sexual harassment is an act that violates someone’s dignity and is of a sexual nature. Some examples of sexual harassment: groping or other unwelcome sexual touches, unwelcome sexual allusions, comments or suggestions, sexualized jokes and the spread of pornographic images or texts*.” Participants answered by responding “Yes” or “No” to the following question: “*Have you during the last year/previous three months experienced that you have been sexually harassed according to the definition above?”.* Consequently, this item corresponds to the respondents subjective interpretation of what kinds of acts should be considered sexual harassment [3].

##### Specific forms of sexual harassment and violence

were measured using six items from the Sexual Experiences Questionnaire (SEQ) [2] that were modified for the Swedish context (Table 1). All items were answered on a five-point Likert scale from “Never” to “Very often”. The items covered: (1) offensive sexual remarks, (2) unwanted sexual attention, (3) presentation or distribution of sexist material, (4) uncomfortable touching, (5) beeing offered benefits for sex and (6) sex against ones will. The SEQ has shown adequate criterion validity and internal consistency (Cronbach’s α = 0.92) in student samples when used as a full scale [27]. In the current analyses each item was instead treated as a *separate exposure*, with exposure defined as responding “Once”, “Sometimes”, “Often”, or “Very often”, and non-exposure as responding “Never”, for each item respectively (Table 1). By using single items rather than scale scores, we can estimate the specific associations between exposure to different forms of SHV and symptoms of depression and anxiety, which would not be possible when using composite score [28, 29]. However, the downside of this approach is that the psychometric properties of individuals item of the SEQ are not known.

**Table 1:** Items used to assess exposure to sexual harassment and violence

Label	Item
	*How often during the [last year/previous three months]*^*a*^*have you experienced that*:
Sexual harassment (wide subjective definition)	You have been sexually harassed according to the definition above? ^b^
*Specific forms of sexual harassment and violence*	
Offensive sexual remarks	Someone made crude and offensive sexual remarks, either publicly (e.g., on the internet or campus) or in private? ^c^
Unwanted sexual attention	Someone gave you unwanted sexual attention (e.g., given you looks or taken other actions that implies sexual interest)? ^c^
Presentation or distribution sexist material	Someone displayed, used, or distributed sexist or suggestive materials (e.g., pictures, stories, or pornography)? ^c^
Uncomfortable touching	Someone touched you in a way that made you feel uncomfortable? ^c^
Offered benefits for sex	Someone implied benefits or better treatment if you were sexually cooperative? ^c^
Sex against ones will	Someone had sex with you against your will? ^c^

^a^ The time-frame of the questions referred to last year in the baseline survey and last three months in the follow-up surveys

^b^ Response categories (coding): No (0), Yes (1)

^c^ Response categories (coding): Never (0), Once (1), Sometimes (1), Often (1), Very often (1)

#### Outcomes

Depression and anxiety symptoms were measured using the short-form Depression, Anxiety and Stress Scale (DASS-21) [30]. The scale consists of 21 items covering the three subscales Depression, Anxiety and Stress, with seven items each. The items are rated on a four-point scale from 0 (“Did not apply to me at all”) to 3 (“Applied to me very much, or most of the time”). The items of each subscale are summed to give subscale scores ranging 0–21. We used only the Depression and Anxiety subscales. DASS-21 has shown good psychometric properties among Swedish university students [31]. In the current sample, Cronbach’s alpha at pre-baseline was 0.91 for the Depression subscale and 0.79 for the Anxiety subscale.

#### Potential confounders

Covariates were selected based on previous literature [4, 7, 12, 13]. We used covariates measured at pre-baseline to ensure that none were on the causal pathway between the SHV exposures and the outcomes. We adjusted for the same covariates in all analyses; pre-baseline levels of all SHV exposures (dichotomous) and depression and anxiety symptoms (continuous), age (continuous), alcohol risk use (low, moderate or high risk alcohol use as defined by the Alcohol, Smoking and Substance Involvement Screening Test [32]), highest level of parental education (university level or below university level), place of birth (Sweden, Nordic countries, Europe, Outside Europe), study year (1st, 2nd, 3rd, > 3rd), education type (technical, medical/health, social science/humanities, economic, other) and civil status (single or partner). By adjusting for prior exposure and outcome levels, we aimed to separate the effect of recent exposure from that of prior exposure and prior outcome levels (Fig. 2). This approach may also reduce potential confounding from prior SHV, and depression and anxiety symptoms present before enrolment in the study (Fig. 2). With adjustment for prior exposure, estimates represents how changes in the exposure are associated with changes in the outcome [19, 33], such that it can be interpreted as the effect of incident rather than prevalent exposure [19]. It should be noted that the estimates aim at the effect of *recent* exposure, not to the *accumulated* effect of repeated exposure to SHV.

### Statistical analyses

Pre-baseline characteristics of the sample for women and men are presented in Table 2, as number and percentages or means and standard deviations (SD). Numbers of exposed to different forms of SHV at baseline is presented in Table 3 for women and men respectively.

The association between the SHV exposures and levels of depression and anxiety symptoms were estimated using Generalized Estimating Equations (GEE). We built separate models for each exposure-outcome combination, for women and men, respectively. All models were specified with independent working correlation structures, identity link functions and robust sandwich standard error estimators. Follow-up time was included in the models as a categoric variable with three levels (FU3, FU6 and FU9), and we included an interaction term between the exposure and follow-up time to let the association between exposure and outcome vary by the length of follow-up. This modelling approach is analogous to performing separate linear regression models for the outcomes at each follow-up time but has the advantage of providing a statistical test of whether the associations between exposure and the outcomes differs depending on the length of the follow-up.

All models were adjusted for pre-baseline covariates. For covariates assumed to have time-varying associations with the outcomes (i.e. pre-baseline SHV and depression and anxiety symptoms), we also included interaction terms with follow-up time.

Results are presented as the adjusted estimated mean differences (MD’s) of depression and anxiety symptoms between exposed and unexposed at each follow-up time, along with 95% confidence intervals (CI’s) (Table 3). For FU6 and FU9, where the estimated mean difference was the sum of the estimate for the exposure coefficient and the coefficient for its interaction with follow-up time, CI’s were calculated according to the formula provided by Figueiras, Domenech-Massons and Cadarso [34].

### Sensitivity analysis

We had complete data on covariates and exposures for all 3503 participants, but data for the outcomes were missing for participants that were lost to follow-up (Fig. 1). GEEs with missing data are unbiased only if data is missing completely at random (MCAR). To assess the sensitivity of our estimates to the MCAR assumption, we imputed data for missing outcomes using multiple imputation (MI) by chained equations, creating 5 imputed datasets. Imputed values were predicted by all covariates and outcomes with a point-biserial correlation to missingness of at least 0.1, using predictive mean matching. All analyses were performed as described above in all imputed datasets, and estimates were pooled using Rubin’s rules (Online resource eTables 3 and 4). MI allows for the less restrictive missing at random assumption (i.e., that outcome data is missing at random conditional on the variables used to predict imputed values) and provides a sensitivity analysis for potential selection bias due to violations of the MCAR assumption.

## Results

We included 2229 women with a mean age of 25.2 years (SD 6.5) at enrolment. The majority were born in Sweden (80%) and were studying at medical education programmes (58%). We also included 1274 men with a mean age of 23.9 years (SD 5.6) at enrolment. The majority were born in Sweden (81%) and were studying at technical education programmes (59%). The one-year prevalence of all forms of SHV were higher among women than men at pre-baseline, as were mean symptoms of depression and anxiety (Table 2).

**Table 2:** Participant characteristics at pre-baseline for women and men

	Women (*n* = 2229)	Men (*n* = 1274)
Age, mean (SD)	25.3 (6.5)	23.9 (5.6)
Type of education, n (%)		
Medical/Health	1293 (58)	383 (30)
Technical	654 (29)	745 (59)
Social science/Humanities	205 (9)	87 (7)
Economic	45 (2)	40 (3)
Other	32 (1)	19 (2)
Highest parental education level = University, n (%)	1599 (72)	940 (74)
Civil status = Single, n (%)	1090 (49)	866 (68)
Place of birth, n (%)		
Sweden	1783 (80)	1026 (81)
Scandinavia	91 (4)	27 (2)
Europe	121 (5)	68 (5)
Outside Europe	234 (11)	153 (12)
Year of studies, n (%)		
1st	935 (42)	483 (38)
2nd	508 (23)	259 (20)
3rd	349 (16)	212 (17)
=>4	437 (20)	320 (25)
Alcohol risk score, n (%)		
Low	1948 (87)	1106 (87)
Moderate	260 (12)	159 (13)
High	21 (1)	9 (1)
SHV^a^, n (%)		
Sexual harassment (wide subjective definition)	641 (29)	59 (5)
Offensive sexual remarks	632 (28)	74 (6)
Unwanted sexual attention	1224 (55)	156 (12)
Presentation or distribution of sexist material	144 (7)	42 (3)
Uncomfortable touching	732 (33)	122 (10)
Offered benefits for sex	111 (5)	19 (2)
Sex against ones will	102 (5)	17 (1)
Depression score, mean (SD)	4.9 (4.7)	4.4 (4.6)
Anxiety score, mean (SD)	3.4 (3.6)	2.2 (2.7)

^a^ The recall time for SHV experiences at pre-baseline was one year

The three-month prevalence of exposure to sexual harassment (wide subjective definition) at baseline was 9.1% (95% CI: 7.9%; 10.4%) among women and 1.2% (95% CI: 0.7%; 1.9%) among men. The three-month prevalence of the specific forms of SHV are presented in Table 3.

Adjusted MDs of depression and anxiety symptoms between exposed and unexposed to SHV at the follow-ups are presented in Table 3. Crude MDs are presented in the Online resource eTables 1 and 2. For women, the adjusted MDs where the CI excluded a null association were: women exposed to sexual harassment (wide subjective definition) had higher adjusted mean depression symptoms at FU3 (MD 1.0 [95% CI: 0.3; 1.7]) and higher anxiety symptoms at FU3 (MD 0.8 [95% CI: 0.3; 1.4]). Women exposed to unwanted sexual attention had higher adjusted mean anxiety symptoms at FU3 (MD 0.5 [95% CI: 0.1; 0.8]) and FU6 (MD 0.4 [95% CI: 0.0; 0.7]). Women exposed to sex against ones will had higher adjusted mean depression symptoms at FU3 (MD 1.7 [95% CI: 0.1; 3.4]), and at FU6 (MD 3.1 [95% CI: 1.0; 5.2]).

For men, the adjusted MDs where the CI excluded the null were as follows: Exposure to sexual harassment (wide subjective definition) was associated with lower mean levels of anxiety symptoms at FU3 (MD -1.2 [95% CI: -1.8; -0.6)). Exposure to unwanted sexual attention was associated to lower mean anxiety levels at FU6 (MD -1.0 [95% CI: -1.8; -0.3]). Men exposed to being offered benefits for sex had lower adjusted mean levels of anxiety (MD -2.7 [95% CI: -4.9; -0.4]) and depression (MD -3.5 [95% CI: -6.9; -0.1]) at FU6. Men exposed to presentation or distribution of sexist material had higher adjusted mean levels of depression symptoms at FU3 (MD 1.8 [95% CI: 0.1;2.6]).

**Table 3:** Adjusted estimates of mean differences of depression and anxiety symptoms between exposed and unexposed to sexual harassment and violence 3 (FU3), 6 (FU6), and 9 (FU9) months after exposure measurement among 2229 women and 1274 men ^a^

	No. exposed (%)	Depression symptoms, Mean difference (95% CI)			Anxiety symptoms, Mean difference (95% CI)		
		FU3	FU6	FU9	FU3	FU6	FU9
*Women*							
Sexual harassment (wide subjective definition)	203 (9)	1.0 (0.3; 1.7)	0.6 (-0.2; 1.3)	0.1 (-0.6; 0.9) ^b^	0.8 (0.3; 1.4)	0.1 (-0.4; 0.6) ^b^	0.2 (-0.4; 0.7) ^b^
Offensive sexual remarks	232 (10)	0.5 (-0.2; 1.2)	0.4 (-0.3; 1.2)	-0.0 (-0.8; 0.7)	0.4 (-0.1; 1.0)	0.2 (-0.3; 0.7)	-0.1 (-0.7; 0.5)
Unwanted sexual attention	548 (25)	0.2 (-0.3; 0.7)	0.2 (-0.3; 0.7)	0.3 (-0.3; 0.8)	0.5 (0.1; 0.8)	0.4 (0.0; 0.7)	0.2 (-0.2; 0.6)
Presentation or distribution of sexist material	53 (2)	0.6 (-0.6; 1.7)	-0.0 (-1.3; 1.3)	-0.5 (-1.9; 0.9)	0.9 (-0.1; 2.0)	0.5 (-0.5; 1.6)	0.2 (-0.9; 1.2)
Uncomfortable touching	199 (9)	0.3 (-0.4; 1)	0.0 (-0.7; 0.7)	-0.5 (-1.2; 0.2)	0.2 (-0.3; 0.7)	0.1 (-0.4; 0.6)	-0.2 (-0.7; 0.4)
Offered benefits for sex	42 (2)	0.9 (-0.5; 2.3)	0.2 (-1.4; 1.9)	-0.3 (-1.7; 1.1)	0.7 (-0.3; 1.6)	-0.1 (-1.2; 1.1)	-0.2 (-1.4; 0.9)
Sex against ones will	22 (1)	1.7 (0.1; 3.4)	3.1 (1.0; 5.2)	1.7 (-0.5; 3.8)	1.3 (-0.1; 2.7)	1.3 (-0.3; 3.0)	0.8 (-1.0; 3.0)
*Men*							
Sexual harassment (wide subjective definition)	15 (1)	-0.4 (-2.0; 1.3)	1.8 (-0.5; 4.0) ^b^	0.1 (-3.1; 3.3)	-1.2 (-1.8; -0.6)	-0.3 (-1.5; 0.9)	-0.4 (-1.8; 1.0)
Offensive sexual remarks	29 (2)	0.3 (-1.3; 2.0)	-1.0 (-2.4; 0.3)	-0.1 (-2.0; 1.7)	0.6 (-0.4; 1.7)	-1.4 (-2.3; -0.6) ^b^	0.4 (-1.1; 2.0)
Unwanted sexual attention	69 (5)	-0.1 (-1.1; 0.9)	-0.3 (-1.4; 0.8)	0.3 (-1.0; 1.6)	-0.8 (-1.5; 0.0)	-1.0 (-1.8; -0.3)	0.3 (-0.7; 1.3) ^b^
Presentation or distribution of sexist material	22 (2)	1.8 (0.1; 3.6)	0.5 (-1.6; 2.6)	1.4 (-1.0; 3.7)	1.3 (-0.1; 2.7)	0.5 (-1.3; 2.3)	1.4 (-0.8; 3.5)
Uncomfortable touching	26 (2)	0.9 (-0.8; 2.6)	0.5 (-1.4; 2.3)	-0.4 (-2.7; 1.9)	0.5 (-0.6; 1.6)	0.0 (-1.1; 1.0)	0.5 (-1.4; 2.3)
Offered benefits for sex	7 (1)	2.6 (-2.2; 7.5)	-3.6 (-6.9; -0.2) ^b^	2.8 (-2.0; 7.6)	2.7 (-0.9; 6.2)	-2.7 (-4.9; -0.4) ^b^	2.0 (-3.4; 7.4)
Sex against ones will	6 (1)	1.6 (-2.1; 5.4)	-1.7 (-4.9; 1.5)	2.2 (-5.9; 10.2)	1.9 (-2.5; 6.3)	-1.8 (-4.1; 0.4)	4.3 (-3.2; 11.8)

^a^ All estimates are adjusted for pre-baseline levels of all SHV exposures and outcomes and their interaction with time, as well as pre-baseline age, alcohol risk use, highest level of parental education, place of birth, study year, education type and civil status

^b^ Indicates MD’s at FU6 and FU9 that are statistically different from the MD at FU3 at *p* < 0.05

For women, estimates were similar when MI was used to account for missing outcome data. For men, however, none of the estimates had confidence excluding the null when MI was used to account for missing outcome data (Online resource eTables 3 and 4).

## Discussion

We followed a sample of university students to estimate the association between recent exposure to different forms of SHV and symptoms of depression and anxiety symptoms 3, 6 and 9 months later, among women and men respectively.

The three-month exposure prevalence at baseline differed between different forms of SHV ranging from < 1–25%, and exposure to most forms of SHV was more common among women than among men, which is in line with prior findings [4, 27]. For instance, the three-month prevalence of sexual harassment (wide subjective definition) was nine times higher among women than among men, and the prevalence of offensive sexual remarks, unwanted sexual attention and uncomfortable touching were about five times more common among women.

For women, recent exposure to the wide subjective definition of sexual harassment, unwanted sexual attention, and sex against ones will were associated with later depression or anxiety symptoms, after controlling for multiple confounders including prior exposure and outcome levels. The most notable of these was the elevated levels of depression among women exposed to sex against their will. This association was stronger and unlike the other associations it showed no sign of decreasing over the nine-month follow-up.

For the other SHV exposures, estimates are more uncertain with CIs of differences between exposed and unexposed not excluding null associations or associations in the opposite direction. The general trend, however, was that mean depression and anxiety symptoms were higher among exposed than unexposed women at FU3, and that MDs between exposed and unexposed decreased over time. Although uncertain, these results indicate that the association between recent exposure to most forms of SHV and subsequent depression and anxiety symptoms may be most pronounced in temporal proximity to the negative sexual experience. An exception is the associations between sex against ones will and depressive symptoms, that showed no signs of decreasing during the length of the follow-up among women.

Many of the associations we observed were rather weak, but it should be noted that outcomes were measured several months after exposure. Given the observed temporal pattern, it is possible that associations would have been more pronounced with a shorter follow-up time. Importantly, our estimates are also restricted to recent exposures, and does therefore not capture the accumulative effect of repeated exposures over time. To our knowledge, this is the first study of how exposure to different forms of SHV is associated to mental health symptoms at different follow-up lengths.

As noted above, there were some variations in the strength of the associations between different forms of SHV and later depression and anxiety symptoms among women. Judging from the point estimates, sex against ones will showed the strongest association to later depression and anxiety symptoms followed by sexual harassment (wide subjective definition), while uncomfortable touching showed the weakest association. These differences in associational strength are uncertain due to overlapping confidence intervals, but the trend indicates that there may be differences in how strongly different forms of SHV are associated with later depression and anxiety symptoms, which is in line with some prior findings [12, 24, 25]. It is perhaps not surprising that exposure to sex against ones will showed the strongest association to subsequent depression and anxiety among women. Sex against ones will is considered rape, according to Swedish law (The Swedish Criminal Code, Chap. 6, 1§), partly because of its negative consequences for the victim. Interestingly, the most widely defined SHV exposure, which we refer to as “sexual harassment (wide subjective definition)” showed the second strongest association to subsequent mental health and was less prevalent than many of the specific forms of SHV. This relatively low prevalence may seem surprising given that this wide question should contain the more specific forms of SHV. Our results are, however, in line with prior research, showing higher prevalence rates when participants are asked about specific SHV experiences than when asked about experiences of SHV in general [35]. It may be that when asked wider and more general questions about SHV, participants are more likely to remember only the most severe experiences, which would explain the stronger associations to subsequent mental health. A limitation is that, given its wide definition, we do not know what those potentially more severe experiences are. The fact that associations with mental health varies between different forms of SHV points towards the need for more narrowly defined and refined measures of SHV in relation to mental health outcomes.

For men, exposure to several form of SHV was associated with lower levels of anxiety and depression at follow-up in the main analyses, but none of the associations had CI’s excluding null associations when MI was used to account for missing outcome data. Exposure to many forms of SHV were uncommon among men in our sample. Together with a smaller sample size than that for women, this resulted in highly variable estimates. Given the uncertainty of the estimates, we refrain from interpreting the estimates for men, as they seem highly affected by random variability and possibly also by selection bias as indicated by the MI analyses.

From a public health perspective, our results highlight the need to consider prevention of SHV when trying to tackle mental health problems among women university students. A special focus may be directed towards severe forms of SHV, such as exposure to sex against ones will, where associations to subsequent mental health seem more pronounced and sustained over longer times.

## Strengths and limitations

The main strength of this study is that we recruited a large sample of university students and followed them over several time-points. This enabled us to estimate associations between recent exposure to SHV and subsequent mental health at different follow-up lengths, while controlling for multiple potential confounders, including prior SHV and mental health. This design provides stronger evidence for a causal relationship between SHV and mental health than most other prior research [19]. It should be noted, however, that most of the found associations were weak, and we cannot rule out the possibility that they may be explained by residual or unmeasured confounding (e.g. dashed arrows in Fig. 2). It could also be argued that the accumulated effect of repeated exposure to SHV on mental health would be of higher interest than the effect of recent exposure or changes in exposure status [36], since exposure to SHV tends to be repeated over time [15]. We chose to focus on the effect of recent exposure for two reasons: (1) it is less vulnerable to confounding by prior mental health, sometimes referred to as “reverse causation” [19], and (2) it more closely reflects the potential effects of intervening to change levels of SHV. That said, it is important to note that our estimates do not represent the accumulated effect of SHV, which may potentially be closer approximated by the crude associations presented in the Online resource Tables 1 and 2. A second strength is that we, to some extent, were able to separate between specific forms of SHV. The use of more narrowly defined exposures strengthens causal inference [29], and gives more information on what kind of SHV exposures that may impact on mental health. This moves us closer to the consistency criterion of causal inference, which is discussed in more detail elsewhere [29, 37].

The main limitation was that, although we recruited a large sample, CIs for many exposures were wide, especially for the men, leaving some estimates uncertain. Second, we had some loss-to follow-up, which may cause selection bias. MI was used to assess the sensitivity of our results to potential selection bias. These sensitivity analyses indicated that while estimates in the women sample were robust, the estimates for men were not. This further hindered us from making inferences of the potential mental health consequences of SHV among men. Third, although evidence suggest that as a scale, the SEQ has adequate reliability and validity [27], there is no formal psychometric evaluation of the items when used as separate measures. The items have face validity, but we cannot rule out that misclassification may have biased our results and led to an attenuation of the associations. Fourth, it has been noted that adjustment for prior exposure and outcomes may increase bias introduced by measurement error which could further have attenuated the associations [36]. Fifth, we tested a large number of associations which increase the risk that some of the findings may be due to random variability (i.e. type-I errors). We believe that this fine-grained analysis was needed to provide nuanced information on the potentially heterogeneous and time-varying effects of exposure to different forms of SHV on mental health, and decided not to adjust for these multiple comparisons since it would inflate the risk of type-II errors [38]. Finally, although we recruited a large sample, most who were invited did not participate in our study and the sample was mainly comprised of students at medical and technical education programs. Our sample is therefore not representative of students overall. Although we see no obvious reason for why the observed associations between exposures and the outcome would be specific to our sample, it is possible that the results do not generalize to other student populations.

## Conclusions

Recent exposure to several forms of SHV was associated with higher subsequent depression and anxiety symptoms among women. Trends indicated that the potential mental health impact of most forms of SHV decreased over time, and association with subsequent mental health differed between specific forms of SHV.

## Electronic supplementary material

Below is the link to the electronic supplementary material.

Supplementary Material 1
