# Supplementary material for: Sexual harassment, sexual violence and subsequent depression and anxiety symptoms among Swedish university students: a cohort study
Source: Soc Psychiatry Psychiatr Epidemiol. 2024 Jun 26;59(12):2313–22. doi: 10.1007/s00127-024-02688-0 (PMC11522111; doi:10.1007/s00127-024-02688-0)
Supplement: Supplementary file 1 — Supplementary Material 1 [file 127_2024_2688_MOESM1_ESM.pdf]

SUPPLEMENTAL MATERIAL

Johansson, F., Edlund, K., Sundgot-Borgen, J., Björklund, C., Côté, P., Onell, C., Sundberg, T. & Skillgate, E. (2023). Sexual Harassment, Sexual Violence and Subsequent Depression and Anxiety Symptoms among Swedish University Students: A Cohort Study. Social Psychiatry and Psychiatric Epidemiology.

Corresponding author: Fred Johansson. Department of Health Promotion Science, Sophiahemmet University, Stockholm, Sweden. Email: fred.johansson@shh.se

eTable 1. Crude mean differences of depression and anxiety symptoms between exposed and unexposed 3, 6, and 9 months after exposure measurement among women students

**Exposure**	**No. exposed (%)**	**Depression symptoms,** Mean difference (95% CI)			**Anxiety symptoms,** Mean difference (95% CI)		
		**FU3**	**FU6**	**FU9**	**FU3**	**FU6**	**FU9**
Sexual harassment - subjective definition	203 (9)	2.2 (1.3; 3.1)	1.6 (0.8; 2.5)	1.3 (0.4; 2.2)	1.4 (0.8; 2)	0.8 (0.2; 1.4)	0.9 (0.2; 1.5)
Offensive sexual remarks	232 (10)	1.9 (1.1; 2.7)	1.6 (0.7; 2.4)	1.2 (0.4; 1.9)	1.4 (0.8; 2)	1.1 (0.5; 1.7)	0.8 (0.2; 1.4)
Unwanted sexual attention	548 (25)	1.1 (0.6; 1.6)	0.8 (0.3; 1.4)	0.9 (0.4; 1.5)	1.1 (0.7; 1.5)	0.9 (0.5; 1.3)	0.7 (0.3; 1.1)
Presentation or distribution of sexist material	53 (2)	1.5 (0; 2.9)	0.7 (-0.8; 2.3)	0.2 (-1.3; 1.8)	1.6 (0.4; 2.7)	1.3 (0.2; 2.4)	0.7 (-0.3; 1.8)
Uncomfortable touching	199 (9)	1.5 (0.7; 2.4)	1.1 (0.3; 1.9)	0.6 (-0.2; 1.3)	1.1 (0.5; 1.7)	0.9 (0.3; 1.5)	0.6 (0; 1.2)
Offered benefits for sex	42 (2)	3 (0.9; 5.1)	2.2 (0.2; 4.1)	1.4 (-0.6; 3.4)	1.8 (0.5; 3)	1.1 (-0.2; 2.4)	0.7 (-0.7; 2.1)
Sex against ones will	22 (1)	4.1 (1.6; 6.6)	4.9 (2.1; 7.8)	3.2 (0.5; 5.8)	2.9 (1; 4.9)	2.7 (0.6; 4.8)	2 (0.4; 3.6)

eTable 2. Crude mean differences of depression and anxiety symptoms between exposed and unexposed 3, 6, and 9 months later among men students

Exposure	No. exposed (%)	Depression symptoms, Mean difference (95% CI)			Anxiety symptoms, Mean difference (95% CI)		
		**FU3**	**FU6**	**FU9**	**FU3**	**FU6**	**FU9**
Sexual harassment - subjective definition	15 (1)	0.4 (-1.9; 2.7)	3.0 (0.1; 5.8)	0.3 (-2.6; 3.2)	0 (-1.1; 1.1)	0.9 (-0.5; 2.3)	0.3 (-1.2; 1.9)
Offensive sexual remarks	29 (2)	0.2 (-1.7; 2.1)	-0.1 (-2; 1.8)	-0.5 (-2.4; 1.3)	1.2 (-0.1; 2.5)	-0.4 (-1.3; 0.4)	0.8 (-0.6; 2.2)
Unwanted sexual attention	69 (5)	0.3 (-0.9; 1.6)	0.7 (-0.7; 2.1)	0.3 (-1.1; 1.6)	0.3 (-0.5; 1.1)	0.1 (-0.6; 0.8)	0.9 (0; 1.8)
Presentation or distribution of sexist material	22 (2)	2.1 (0.1; 4)	0.4 (-2.2; 3.1)	1.3 (-1.1; 3.8)	2 (0.3; 3.7)	0.9 (-1.2; 2.9)	2.1 (-0.1; 4.2)
Uncomfortable touching	26 (2)	1.7 (-0.3; 3.7)	1.6 (-0.8; 3.9)	0.5 (-2; 2.9)	1.3 (-0.1; 2.6)	0.6 (-0.7; 1.8)	0.7 (-0.9; 2.4)
Offered benefits for sex	7 (1)	1.8 (-3.7; 7.3)	-2.8 (-4.3; -1.2)	0.6 (-4.2; 5.3)	3 (-1.1; 7.1)	-1.2 (-2.3; -0.2)	1.8 (-2.7; 6.4)
Sex against ones will	6 (1)	2.5 (-0.5; 5.4)	-2.2 (-4.3; -0.1)	0.8 (-6.5; 8)	3 (-1.1; 7.2)	-1.5 (-2; -1)	3.7 (-3.1; 10.5)

eTable 3. Sensitivity analysis using multiple imputation. Adjusted mean differences of depression and anxiety symptoms between exposed and unexposed 3, 6, and 9 months after exposure measurement among women students

**Exposure**	**No. exposed (%)**	**Depression symptoms,** Mean difference (95% CI)			**Anxiety symptoms,** Mean difference (95% CI)		
		**FU3**	**FU6**	**FU9**	**FU3**	**FU6**	**FU9**
Sexual harassment - subjective definition	203 (9)	0.9 (0.2; 1.6)	0.5 (-0.3; 1.3)	0.3 (-0.5; 1.1)	0.7 (0.2; 1.3)	0.2 (-0.4; 0.8)	0.3 (-0.4; 0.9)
Offensive sexual remarks	232 (10)	0.5 (-0.2; 1.2)	0.5 (-0.4; 1.3)	0.1 (-0.9; 1)	0.4 (-0.1; 0.9)	0.2 (-0.3; 0.8)	0 (-0.6; 0.7)
Unwanted sexual attention	548 (25)	0.2 (-0.3; 0.7)	0.2 (-0.3; 0.8)	0.1 (-0.4; 0.7)	0.4 (0.0; 0.7)	0.3 (-0.1; 0.7)	0.1 (-0.3; 0.5)
Presentation or distribution of sexist material	53 (2)	0.5 (-0.7; 1.6)	0 (-1.5; 1.4)	-0.2 (-1.7; 1.2)	0.9 (-0.1; 1.9)	0.4 (-0.7; 1.5)	0.1 (-1.0; 1.2)
Uncomfortable touching	199 (9)	0.3 (-0.4; 1)	0.2 (-0.8; 1.1)	-0.2 (-1.1; 0.8)	0.1 (-0.4; 0.6)	0.1 (-0.6; 0.7)	-0.1 (-0.8; 0.7)
Offered benefits for sex	42 (2)	0.8 (-0.7; 2.3)	0.1 (-1.8; 2)	-0.3 (-2.2; 1.6)	0.4 (-0.6; 1.4)	-0.2 (-1.0.4; 1)	-0.4 (-1.6; 0.9)
Sex against ones will	22 (1)	1.6 (-0.3; 3.5)	2.9 (0.4; 5.3)	1.8 (-0.6; 4.3)	1.2 (-0.2; 2.6)	1.3 (-0.3; 3)	0.8 (-0.9; 2.6)

eTable 4. Sensitivity analysis using multiple imputation. Adjusted mean differences of depression and anxiety symptoms between exposed and unexposed 3, 6, and 9 months later among men students

**Exposure**	**No. exposed (%)**	**Depression symptoms,** Mean difference (95% CI)			**Anxiety symptoms,** Mean difference (95% CI)		
		**FU3**	**FU6**	**FU9**	**FU3**	**FU6**	**FU9**
Sexual harassment - subjective definition	15 (1)	-0.4 (-2.1; 1.3)	1.3 (-0.9; 3.5)	0.3 (-2.3; 2.9)	-1.1 (-1.7; -0.5)	-0.2 (-1.5; 1.2)	0 (-1.2; 1.3)
Offensive sexual remarks	29 (2)	0.2 (-1.4; 1.8)	-1.0 (-2.5; 0.4)	-0.4 (-2.2; 1.4)	0.6 (-0.5; 1.7)	-1.1 (-2.2; 0)	0.5 (-0.9; 1.9)
Unwanted sexual attention	69 (5)	0.2 (-0.9; 1.4)	0.3 (-1.4; 1.9)	0.4 (-1.1; 2.0)	-0.6 (-1.3; 0.2)	-0.5 (-1.4; 0.5)	0.5 (-0.5; 1.5)
Presentation or distribution of sexist material	22 (2)	1.7 (-0.1; 3.5)	0.6 (-1.7; 2.9)	1.2 (-1.4; 3.8)	1.3 (-0.1; 2.7)	0.5 (-1.4; 2.4)	1.3 (-1.0; 3.6)
Uncomfortable touching	26 (2)	0.7 (-1; 2.3)	1 (-0.9; 2.9)	0.4 (-1.8; 2.6)	0.4 (-0.8; 1.6)	0.4 (-1.1; 1.8)	0.9 (-0.9; 2.6)
Offered benefits for sex	7 (1)	2.1 (-2.5; 6.7)	-1.9 (-6.6; 2.8)	2.0 (-3.3; 7.3)	2.4 (-1; 5.7)	-1.2 (-4.9; 2.5)	2.3 (-2.3; 7.0)
Sex against ones will	6 (1)	1.1 (-2.6; 4.8)	-1.1 (-6.3; 4.2)	0.9 (-5.6; 7.5)	1.6 (-2.3; 5.6)	-1.1 (-5.6; 3.4)	2.7 (-2.8; 8.1)
